# The emerging roles of non-coding competing endogenous RNA in hepatocellular carcinoma

**DOI:** 10.1186/s12935-020-01581-5

**Published:** 2020-10-12

**Authors:** Gang Xu, Wei-Yu Xu, Yao Xiao, Bao Jin, Shun-Da Du, Yi-lei Mao, Zhong-Tao Zhang

**Affiliations:** 1 Department of Liver Surgery, Peking Union Medical College (PUMC) Hospital and Chinese Academy of Medical Sciences, 1# Shuaifuyuan, Wangfujing, Dong-Cheng District, Beijing, 100730 China; 2 Department of General Surgery, Beijing Friendship Hospital, Capital Medical University; Beijing Key Laboratory of Cancer Invasion and Metastasis Research & National Clinical Research Center for Digestive Diseases, No. 95 Yong-An Road, Xi-Cheng District, Beijing, 100050 People’s Republic of China

**Keywords:** Hepatocellular carcinoma, Noncoding RNA, Competing endogenous RNA

## Abstract

Accumulating evidence has emerged revealing that noncoding RNAs (ncRNAs) play essential roles in the occurrence and development of hepatocellular carcinoma (HCC). However, the complicated regulatory interactions among various ncRNAs in the development of HCC are not entirely understood. The newly discovered mechanism of competing endogenous RNAs (ceRNAs) uncovered regulatory interactions among different varieties of RNAs. In recent years, a growing number of studies have suggested that ncRNAs, including long ncRNAs, circular RNAs and pseudogenes, play major roles in the biological functions of the ceRNA network in HCC. These ncRNAs can share microRNA response elements to affect microRNA affinity with target RNAs, thus regulating gene expression at the transcriptional level and both physiological and pathological processes. The ncRNAs that function as ceRNAs are involved in diverse biological processes in HCC cells, such as tumor cell proliferation, epithelial-mesenchymal transition, invasion, metastasis and chemoresistance. Based on these findings, ncRNAs that act as ceRNAs may be promising candidates for clinical diagnosis and treatments. In this review, we discuss the mechanisms and research methods of ceRNA networks. We also reviewed the recent advances in studying the roles of ncRNAs as ceRNAs in HCC and highlight possible directions and possibilities of ceRNAs as diagnostic biomarkers or therapeutic targets. Finally, the limitations, gaps in knowledge and opportunities for future research are also discussed.

## Background

Hepatocellular carcinoma (HCC), the most prevalent subtype of hepatic malignancy, is one of the leading causes of cancer-associated mortality worldwide [1]. There are an estimated 62 million new cases of HCC annually, 85% of which are diagnosed in developing countries [2]. Owing to the insidious symptoms and early metastases, most HCC patients are diagnosed at an advanced stage, leading to limited efficacy and even ineffectiveness of therapeutic approaches [3]. Despite continuous therapeutic advances, such as in surgical resection, liver transplantation and radiofrequency ablation, the 5-year survival rate for HCC patients is still under 20% [4, 5]. Therefore, it is important to identify the underlying mechanism as well as precise diagnostic and prognostic biomarkers for early diagnosis and risk assessment. Furthermore, identifying effective therapeutic targets for HCC is critical.

**Fig. 1:**
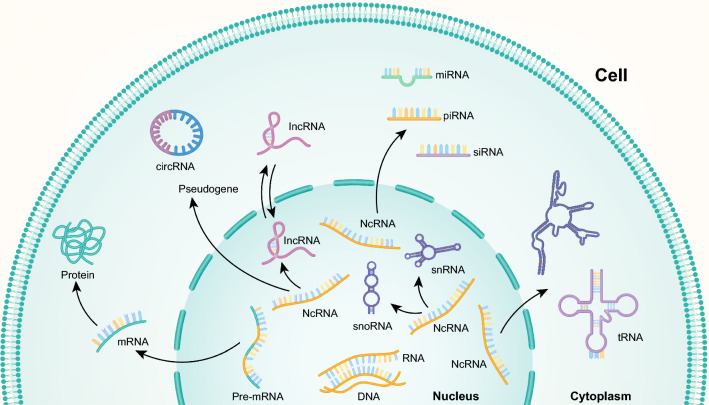
The classification of coding and noncoding RNA. Eukaryotic mRNA molecules are usually composed of small segments of the original gene and are generated by a process of cleavage and rejoining from an original precursor RNA (pre-mRNA) molecule, which is an exact copy of the gene. Noncoding RNA (ncRNA) mainly include long non-coding RNA (lncRNA), microRNA (miRNA), pseudogene, circular RNA (circRNA), small interfering RNA (siRNA), piwi-interacting RNA (piRNA), small nucleolar RNA (snoRNA), small nuclear RNA (snRNA), ribosomal RNA (rRNA) and transfer RNA (tRNA)

Carcinogenesis is a complicated multi-step, multi-stage process involving both genetic and epigenetic alterations [3]. However, the molecular pathogenesis of HCC remains unclear. Furthermore, the majority of previous studies that examined the molecular mechanisms of hepatic tumorigenesis have focused on protein-coding genes and not yet provided comprehensive and detailed mechanisms of HCC. High-throughput sequencing technology has revealed numerous large and small non-coding RNAs (ncRNAs) that are critically involved in carcinogenesis [6]. NcRNAs, which universally exist in a broad range of organisms, are a group of RNAs without protein-coding functions and include long non-coding RNA (lncRNA), microRNA (miRNA), pseudogene, circular RNA (circRNA), small interfering RNA, small nucleolar RNA, ribosomal RNA and transfer RNA (Fig. 1). LncRNA, miRNA, pseudogene and circRNA are primarily involved in post-transcriptional regulation [7]. Recently, accumulating evidence has demonstrated that these ncRNAs are potential diagnostic and prognostic biomarkers for HCC [8–10]. Nevertheless, the specific functions and mechanisms of most ncRNAs in HCC remain unclear.

Recent studies have reported competing endogenous RNA (ceRNA) regulatory networks (ceRNET), in which ceRNAs modulate each other at the post-transcriptional level via competition of shared miRNAs. Functionally, ceRNA networks are considered the bridge to connect the functions of protein-coding mRNAs with the functions of ncRNAs, including lncRNAs, circRNAs, miRNAs and pseudogenes. According to the ceRNA hypothesis, ceRNAs sharing miRNA response elements (MREs) may affect miRNA affinity with mRNAs, thereby triggering gene silencing [11]. Because all transcripts with MREs could theoretically serve as ceRNAs, these transcripts may be considered as universal regulators of post-transcriptional events under physiological and pathological conditions. Aberrantly expressed ceRNAs may cause dysregulation of ceRNA networks, leading to human diseases, including cancer [12–14].

**Fig. 2:**
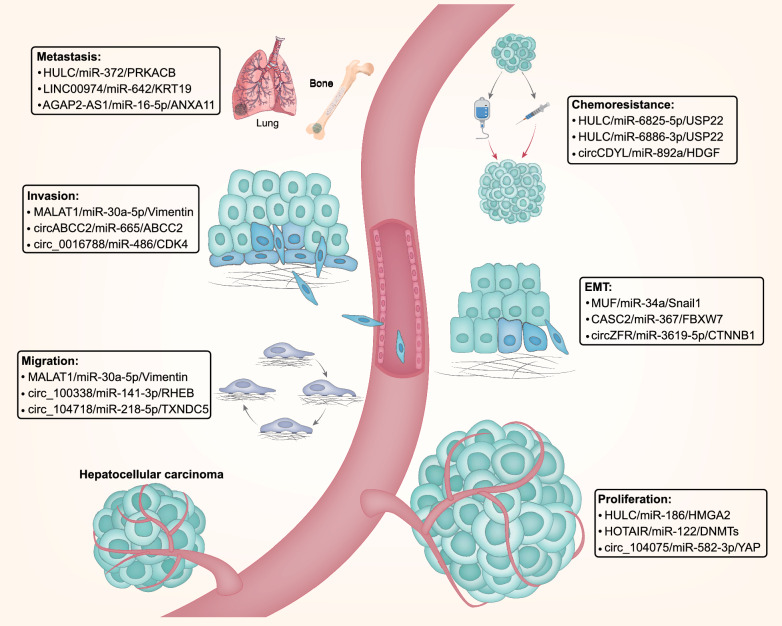
Summary of ncRNAs act as ceRNAs mediated functions. NcRNAs can regulate hepatocellular carcinoma progression, such as cell proliferation, migration, epithelial–mesenchymal transition (EMT), invasion, distant metastasis and chemotherapy resistance

Several reports have demonstrated the roles of ncRNAs as ceRNAs in multiple processes of pathogenesis in HCC (Fig. 2). Based on the demonstrated function of ncRNAs as ceRNAs in HCC, ncRNAs may serve as potential biomarkers and therapeutic targets. In this review, we will discuss ncRNAs that function as ceRNAs in HCC and their relevance to current clinical practice. We first summarize the mechanisms and research methods of ceRNA and ceRNETs and provide some examples of the far-reaching roles for these molecules in affecting HCC processes. We then discuss how the basic science insights into the function of ncRNAs as ceRNAs are being applied to develop next-generation biomarkers and therapies in HCC. As the so-called “dark matter” of the genome continues to be brought into the light, it is evident that targeting ncRNAs that function as ceRNAs and ceRNETs has great potential to impact HCC patient care.

## Mechanisms and research methods of ceRNA and ceRNETs

**Fig. 3:**
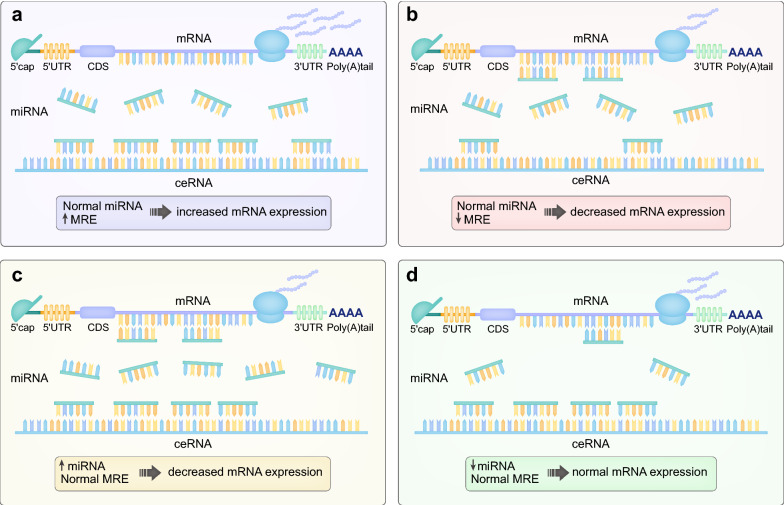
The mechanisms of ceRNA regulatory networks. CeRNAs and mRNAs share a pool of miRNAs, and the competition for miRNAs leads to a dynamic regulation of the expression level of mRNAs. **a** ceRNA with up-regulated miRNA response elements (MREs) can “absorb” more miRNAs, thus the mRNA expression is increased. **b** When the MRE is down-regulated, miRNAs tend to bind with and silence mRNAs, leading to a decrease in mRNA expression. **c** miRNA up-regulation exceeds the “sponge effect” of ceRNAs, and mRNA expression is inhibited by excessive miRNAs. **d** miRNA level is decreased, and mRNA can be expressed normally. In addition, the subtle regulation of one certain mRNA can be achieved by the interactions of multiple miRNAs and ceRNAs

Our expanded understanding of the transcriptome has led to the identification of diverse MREs in various RNA transcripts. The ceRNA hypothesis, which was initially established in 2011 [15], proposes that RNAs that share MREs can function as ceRNAs and lead to an impairment of miRNA activity (Fig. 3) [11, 16]. The upregulated expression of one particular transcript could sequestrate more miRNA copies from the miRNA pool and subsequently derepress other transcripts and vice versa. Moreover, the existence of MREs in lncRNAs, circRNAs and pseudogenes has suggested a modulation and control among these ncRNAs through a complicated ceRNET [17].

The in-depth studies on ceRNA interactions have revealed several potential factors for optimal ceRNA activity. First, the interaction can be affected by the abundance and relative concentration of ceRNAs and interacting miRNAs. Studies have reported that ceRNA activity reaches an optimal state when the levels of miRNA and ceRNA are nearly equimolar [18, 19]. CeRNAs have limited sequestration ability on highly abundant miRNAs; however, lowly expressed miRNAs exhibit minimal interaction with active ceRNAs due to their restricted number. Second, the location of ceRNA components is another potential factor. The expression of miRNAs is cell- and tissue-specific, and thus some ceRNAs only function in specific cells with shared accessible miRNAs [20]. Moreover, the subcellular localization of ceRNAs may also impact the availability of ceRNAs to shared miRNAs, thereby affecting ceRNA activity [21]. Third, since each miRNA can competitively bind to multiple transcripts, the binding affinities of these transcripts to miRNAs can impact the competing effects; in other words, higher affinity indicates more potent competition [22]. Of note, the binding affinity between target RNAs and miRNAs is primarily affected by the binding of MREs (located in target RNA) with seed regions (located in miRNA). Binding is additionally influenced by single-nucleotide polymorphisms, RNA editing and alternative splicing [23–26], as these factors can alter the seed regions and the target spectrum, which further lead to the production, degradation or alteration of miRNA binding sites. Fourth, the abundance of argonaute (Ago), which is the catalytic component of the RNA-induced silencing complex, is likely to be a rate-limiting factor for ceRNA crosstalk. The seed region in the RNA-induced silencing complex forms base pairs with the MREs located within the 3′-untranslated region of target RNA to modulate target RNA [27]. Finally, RNA transcripts are also likely to compete for shared RNA binding proteins, which are involved in RNA degradation, stability and splicing [28], in addition to shared miRNAs. This could hinder miRNA-target binding via MRE occupancy or inversely promote miRNA-target binding via the recruitment of miRNAs to target RNA, implicating their involvement in the ceRNET [29, 30].

**Fig. 4:**
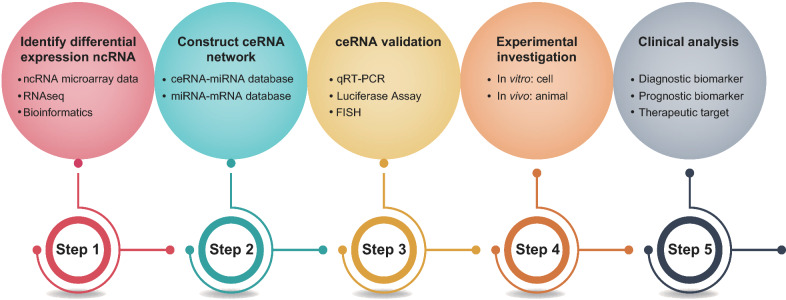
The steps and research methods for studying competing endogenous RNA network in HCC. First, differentially expressed ncRNAs in HCC should be obtained using various resources and software. Second, computer algorithms and public databases may be used to predict interactions between different transcripts and to construct ceRNET. Third, ceRNA interactions within ceRNET should be validated experimentally. Fourth, the dysregulation of ceRNAs in HCC pathogenesis may be further investigated by functional studies involving cell and animal experiments. Finally, the hub genes within ceRNET should be translated into clinical manegement of HCC. ncRNA: Non-coding RNA; ceRNA: Competing endogenous RNA; ceRNET: ceRNA regulatory network; mRNA: Messenger RNA; miRNA: MicroRNA; qRT-PCR: Quantitative reverse transcription polymerase chain reaction; FISH: Fluorescence in situ hybridization

The prediction, construction and validation of ceRNETs have become possible from the transcriptome data derived from various malignancies and the development of bioinformatics and computational methods. The construction of ceRNETs can effectively predict the function and underlying mechanisms of related ceRNAs. CeRNA crosstalk, a posttranscriptional event, is mediated by miRNAs and dependent on MREs located within each transcript [11]. Therefore, MRE identification in the related transcript of interest plays a decisive role in predicting ceRNA crosstalk. Silicon-based or high-throughput databases have been developed to facilitate the identification of ceRNETs (Table 1). The research methods and processes involving ceRNETs in HCC to date are presented in Fig. 4.

**Table 1:** Summary of computational approaches for identifying the ceRNA networks

Databases	Brief description	Link	Ref
starBase v2.0	Database supplies comprehensive interaction network of ncRNAs (lncRNAs, miRNAs, and ceRNAs), mRNA, and proteins in cancer cells and normal tissues based on 108 CLIP-Seq	http://starbase.sysu.edu.cn/	[31]
NPInter v3.0	Database supplies comprehensive interaction among ncRNAs (except tRNAs and rRNAs), lncRNAs and others. It provides different types of basic information about the interaction	http://www.bioinfo.org/NPInter/	[32]
DIANA-LncBase v2	Database supplies two different miRNA–lncRNA interaction modules. One module is experimentally supported, and the other is in silico predicted interactions	http://www.microrna.gr/LncBase/	[33, 34]
circBase	Database provides scripts to identify known and novel circRNAs in sequencing data.	http://www.circbase.org/	[35]
LNCediting	Database supplies information about RNA editing in lncRNAs and miRNA–lncRNA interactions	http://bioinfo.life.hust.edu.cn/LNCediting/	[36]
CircInteractome	Database supplies information about circRNAs and their interaction with proteins or miRNAs	http://circinteractome.nia.nih.gov/	[37]
Cancer-Specific-CirRNA-Database (CSCD)	Database for cancer-specific circRNAs	http://gb.whu.edu.cn/CSCD/	[38]
spongeScan	Database supplies information about microRNA binding elements in lncRNA sequences	http://spongescan.rc.ufl.edu	[39]
SomamiR 2.0	Database provides information and functional analysis of expected miRNA–ceRNA interaction	http://compbio.uthsc.edu/SomamiR	[40]
lnCeDB	Database provides information of human lncRNA that acts as ceRNAs	http://gyanxet-beta.com/lncedb/	[41]
miRBase	Database provides miRNA gene hunters with unique names for novel miRNA genes prior to publication of results	http://www.mirbase.org/	[42]
Targetscan	Database provides information of predicted microRNA targets	http://www.targetscan.org/vert_72/	[43]
miRcode	Database provides “whole transcriptome” human microRNA target predictions based on the comprehensive GENCODE gene annotation.	http://www.mircode.org/	[44]

ncRNA: noncoding RNA; ceRNA: competing endogenous RNA; lncRNA: long noncoding RNA; mRNAs: messenger RNAs; miRNA: microRNA; circRNA: circularRNA; Ref: reference

## The types of ncRNAs acting as ceRNAs and their possible roles in HCC

### LncRNAs as ceRNAs

LncRNAs, ncRNAs over 200 nt in length that originate from promoter-proximal, antisense and intergenic regions, carry out their regulatory function by interacting with genomic DNA, mRNAs and proteins as well as other ncRNAs [45, 46]. LncRNAs may act as sponges, scaffolds, decoys, signals and guides. LncRNAs can regulate gene expression at epigenetic, transcriptional, posttranscriptional and translational levels. Thus, these ncRNAs are considered to have pleiotropic impacts and considered “master regulators” of the genome [47, 48]. They exert regulatory effects in both physiological and pathological conditions, including in various cancers such as HCC [49–51]. Emerging evidence has revealed that a large family of lncRNAs function as ceRNAs for modulation of the expression and biological features of miRNAs in HCC [48] (Table 2).

**Table 2:** Validated ceRNA networks shaped by the ceRNA function of lncRNAs in HCC

ceRNA network type	ceRNA member	Shared miRNA(s)	Competing target (mRNA)	ceRNA role	Experiments related to mechanism	Related HCC function mechanisms	Ref
LncRNA–miRNA–mRNA	HULC	miR-372	PRKACB	Oncogenic	Cellular	Invasion and metastasis	[12]
		miR-6825-5p, miR-6845-5p, miR-6886-3p	USP22	Oncogenic	shRNA knockdown in mouse xenografts	Chemosensitivity	[52]
		miR-200a-3p	ZEB1	Oncogenic	siRNA knockdown in mouse xenografts	Invasion and EMT	[53]
		miR-186	HMGA2	Oncogenic	Overexpression, antimiRs in mouse xenografts	Proliferation	[54]
	HOTAIR	miR-122	DNMTs	Oncogenic	shRNA knockdown in mouse xenografts	Proliferation	[55]
		miR-23b-3p	ZEB1	Oncogenic	Overexpression in mouse xenografts	Invasion and EMT	[56]
	MALAT1	miR-30a-5p	Vimentin	Oncogenic	shRNA knockdown in mouse xenografts	Migration and invasion	[57]
		miR-204	SIRT1	Oncogenic	Cellular	Migration and invasion	[58]
		miR-143-3p	ZEB1	Oncogenic	Cellular	Proliferation and invasion	[59]
	MIAT	miR-214	EZH2	Oncogenic	siRNA knockdown in mouse xenografts	Proliferation and invasion	[60]
	LINC00974	miR-642	KRT19	Oncogenic	shRNA knockdown in mouse xenografts	Proliferation and metastasis	[61]
	CCAT1	Let-7	HMGA2, c-Myc	Oncogenic	Cellular	Proliferation and migration	[62]
	DANCR	miR-214, miR-320a, miR-199a	CTNNB1	Oncogenic	siRNA knockdown in mouse xenografts	Invasion and metastasis	[63]
		miR-27a-3p	ROCK1, LIMK1, COFILIN1	Oncogenic	shRNA knockdown in mouse xenografts	Proliferation and metastasis	[64]
		miR-216a-5p	KLF12	Oncogenic	shRNA knockdown in mouse xenografts	Migration and invasion	[65]
	HOTTIP	miR-125b	HOXA	Oncogenic	shRNA knockdown in mouse xenografts	Proliferation and migration	[66]
	ATB	miR-200 family	ZEB1, ZEB2	Oncogenic	Overexpression, knockdown in mouse xenografts	Invasion and EMT	[67]
	UCA1	miR-216b	FGFR1	Oncogenic	siRNA knockdown in mouse xenografts	Proliferation and metastasis	[68]
		miR-203	Snail2	Oncogenic	shRNA knockdown in mouse xenografts	Proliferation and invasion	[69]
	FAL1	miR-1236	AFP, ZEB1	Oncogenic	Cellular	Proliferation and migration	[70]
	MUF	miR-34a	Snail1	Oncogenic	overexpression in mouse xenografts	EMT	[71]
	HOXD-AS1	miR-130a-3p	SOX4	Oncogenic	shRNA knockdown in mouse xenografts	Migration and invasion	[72]
	SNHG8	miR-149-5p	PPM1F	Oncogenic	shRNA knockdown in mouse xenografts	Proliferation and metastasis	[73]
	CDKN2B-AS1	let-7c-5p	NAP1L1	Oncogenic	shRNA knockdown in mouse xenografts	Proliferation and metastasis	[74]
	FLVCR1-AS1	miR-513c	MET	Oncogenic	shRNA knockdown in mouse xenografts	Proliferation, migration and invasion	[75]
	ZFAS1	miR-150	ZEB1, MMP14, MMP16	Oncogenic	Overexpression, shRNA knockdown in mouse xenografts	Proliferation and metastasis	[76]
	NEAT1	miR-485	STAT3	Oncogenic	Cellular	Migration and invasion	[77]
	TUG1	miR-142-3p	ZEB1	Oncogenic	shRNA knockdown in mouse xenografts	Proliferation and EMT	[78]
		miR-144	JAK2	Oncogenic	shRNA knockdown in mouse xenografts	Proliferation and migration	[79]
	ANRIL	miR-122-5p	NR	Oncogenic	shRNA knockdown in mouse xenografts	Proliferation and metastasis	[80]
	n335586	miR-924	CKMT1A	Oncogenic	Overexpression in mouse xenografts	Migration and invasion	[81]
	HOXA-AS2	miR-520c-3p	GPC3	Oncogenic	shRNA knockdown in mouse xenografts	Proliferation and EMT	[82]
	PCAT-1	miR-215	CRKL	Oncogenic	shRNA knockdown, miR upregulation in mouse xenografts	Proliferation	[83]
	MCM3AP-AS1	miR-194-5p	FOXA1	Oncogenic	shRNA knockdown in mouse xenografts	Proliferation	[84]
	SNHG6-003	miR-26a/b	TAK1	Oncogenic	Overexpression in mouse xenografts	Proliferation	[85]
	TP73-AS1	miR-200a	HMGB1, RAGE	Oncogenic	Cellular	Proliferation	[86]
	DSCR8	miR-485-5p	FZD7	Oncogenic	Overexpression, shRNA knockdown in mouse xenografts	Proliferation	[87]
	LINC00707	miR-206	CDK14	Oncogenic	shRNA knockdown in mouse xenografts	Proliferation, migration and invasion	[88]
	AGAP2-AS1	miR-16-5p	ANXA11	Oncogenic	Overexpression, shRNA knockdown in mouse xenografts	Proliferation and metastasis	[89]
	miat	miR-22-3p	sirt1	Oncogenic	Knockdown in mouse xenografts	Proliferation	[90]
	DSCAM-AS1	miR-338-3p	CyclinD1, SMO	Oncogenic	shRNA knockdown in mouse xenografts	Proliferation, migration and invasion	[91]
	XIST	miR-194-5p	MAPK1	Oncogenic	shRNA knockdown in mouse xenografts	Proliferation, migration and invasion	[92]
		miR-497-5p	PDCD4	Tumor suppressive	Overexpression in mouse xenografts	Proliferation and metastasis	[93]
		miR-92b	Smad7	Tumor suppressive	Overexpression, knockdown in mouse xenografts	Proliferation and metastasis	[94]
	LINC00657	miR-106a-5p	PTEN	Tumor suppressive	shRNA knockdown in mouse xenografts	Proliferation, migration and invasion	[95]
	MEG3	miR-9-5p	SOX11	Tumor suppressive	Cellular	Proliferation	[96]
	SNHG16	miR-93	NR	Tumor suppressive	Overexpression in mouse xenografts	Cell proliferation and chemosensitivity	[97]
	CASC2	miR-367	FBXW7	Tumor suppressive	Overexpression downregulation in mouse xenografts	EMT	[98]
	MIR31HG	miR-575	ST7L	Tumor suppressive	shRNA knockdown in mouse xenografts	Proliferation and metastasis	[99]
	FTX	miR-374a	WIF1, PTEN, WNT5A	Tumor suppressive	Overexpression in mouse xenografts	Invasion and EMT	[100]

ceRNA: competing endogenous RNA; lncRNA: long noncoding RNA; mRNAs: messenger RNAs; miRNA: microRNA; HCC: hepatocellular carcinoma; EMT: epithelial–mesenchymal transition; Ref: reference

LncRNAs can exert their functions in the HCC pathophysiological process by participating in a certain pathway but through interactions with different miRNAs. The lncRNA metastasis-associated lung adenocarcinoma transcript-1 (MALAT1) promotes HCC migration and invasion by three pathways [57–59]. First, MALAT1 competes with miR-30a-5p to regulate vimentin gene expression. Pan et al. [57] first demonstrated the sponge role of MALAT-1 for miR-30a-5p and described its functions in HCC as a ceRNA. The authors measured MALAT1 and vimentin expression levels in paired HCC and normal adjacent tissues by RT-PCR and predicted potential miR-30a-5p binding sites in MALAT1 and the target site of miR-30a-5p in vimentin using bioinformatic analyses. Co-transfection of plasmids and miR-30a-5p mimics into 293T cells was used to discover how miR-30a-5p regulates MALAT1 expression levels. Dual luciferase assay showed that miR-30a-5p significantly decreased vimentin expression level, and subsequent experiments showed that miR-30a-5p inhibits HCC cell migration and invasion. The role of MALAT1 in HCC cell growth was also tested using in vivo experiments, in which the authors injected HepG2 cells transfected with sh-MALAT1 into nude mice; the tumor volume and size in the shRNA knockdown group were smaller than that of the negative control group. These results provided in vivo evidence that MALAT1 promotes HCC growth. Second, MALAT1 competes with miR-204 to regulate SIRT1 gene expression. Hou et al. [58] used shRNA knockdown and loss-of-function strategies on MALAT1 in HepG2 cells to screen potential MALAT-1-interacting miRNA candidates, and identified miR-204 had shown the best correlation. The authors treated HepG2 cells with miR-204 inhibitors and mimics and found that MALAT-1 level was increased and the migration and invasion abilities of HepG2 cells were enhanced in miR-204 inhibitor group. The results demonstrated that MALAT1 derepresses SIRT1 by sponging and competitively binding to miR-204, causing enhanced migration, invasion and epithelial-mesenchymal transition (EMT) of HCC. Finally, MALAT1 also competes with miR-143-3p for the tumor suppressor ZEB1. Chen et al. [59] conducted a clinicopathological analysis in 56 paired HCC and non-tumor liver samples to explore the association between MALAT-1 expression level and clinical characteristics including age, sex, tumor size, tumor differentiation, TNM stage and distant metastasis. The authors also predicted possible interactions among MALAT1, miR-143-3p and ZEB1 based on a bioinformatics analysis. SiRNA knockdown of MALAT-1 was performed in several cell lines, and Matrigel invasion assays proved that si-MALAT-1 inhibited HCC cell invasion. Subsequent experiments confirmed the existence of the MALAT1/miR-143-3p/ZEB1 regulatory pathway and showed that high expression of MALAT1 in HCC led to increased ZEB1, thus promoting the proliferation and migration of HCC.

**Fig. 5:**
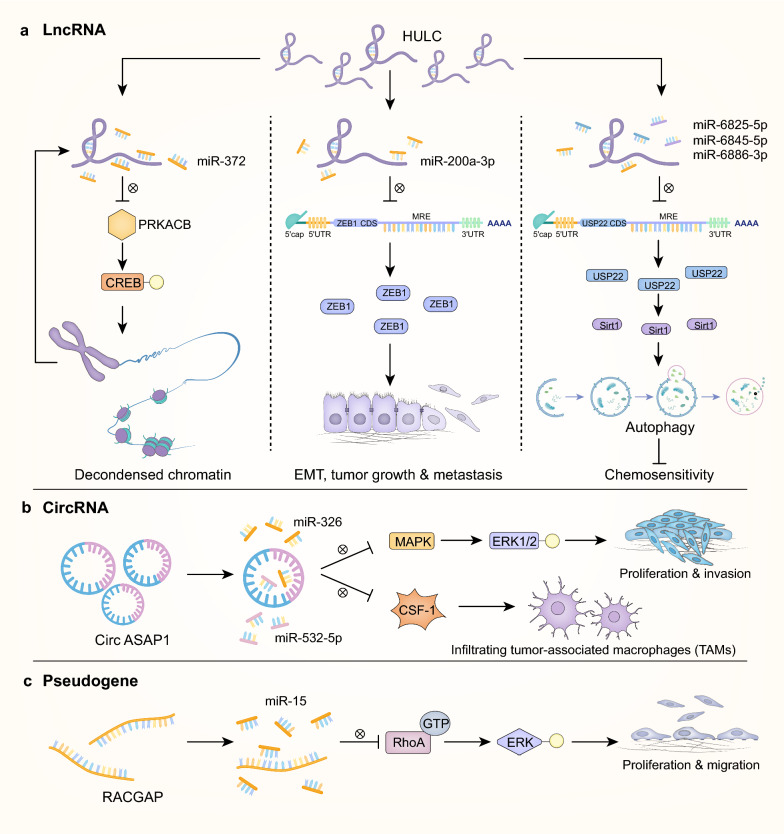
Simplified examples of the roles that different classes of ceRNAs play in HCC oncogenesis. **a** LncRNA: HULC functions in HCC by three mechanisms: (1) Transcription activation of HULC decreases miR-372 level in HCC, derepresses PRKACB function and CREB phosphorylation, hence enhancing chromatin accessibility and transcription. (2) HULC exerts a negative regulatory effect on miR-200a-3p and subsequent upregulation of ZEB1, which facilitates EMT, tumor growth and metastasis. (3) HULC sponges miR-6825-5p, miR-6845-5p and miR-6886-3p, leading to increased expression of USP22 and Sirt1, which activates the protective autophagy pathway and attenuates HCC cell sensitivity to chemotherapeutic agents. **b** CircRNA: CircASAP1 decreases miR-326 and miR-532-5p level, thus promoting proliferation and invasion of HCC through miR-326/MAPK/ERK1/2 signaling, and raising TAMs infiltration through CSF-1 activation and secretion. **c** Pseudogene: RACGAP binds to miR-15, enhances the binding of GTP to RhoA and further ERK phosphorylation, promoting tumor cell proliferation and migration

Several lncRNAs can influence HCC behavior by several distinct mechanisms through interactions with different miRNAs and their corresponding mRNAs. HULC is one of the most upregulated lncRNAs in HCC and was found to function in HCC by at least three mechanisms (Fig. 5a). Wang et al. [12] performed chromatin accessibility by real-time PCR (CHART-PCR) assays to evaluate the accessibility of genomic DNA to nuclease and analyze different regions of open chromatin around the HULC promoter. ChIP assays demonstrated that increased euchromatic histone modifications are correlated with transcription activation of HULC. Subsequent experiments revealed significantly increased miR-372 level after siRNA-mediated inhibition of HULC, indicating that HULC decreases miR-372 expression in HCC and enhances chromatin accessibility and transcription. Li et al. [53] observed that a higher expression level of HULC in HCC tissues is associated with many enhanced EMT features, including histological morphology, physiological behavior and EMT markers such as E-cadherin, N-cadherin, ZO-1, vimentin, β-catenin, Snail and ZEB1. The authors proposed that HULC exhibits a negative regulatory effect on miR-200a-3p and upregulates ZEB1, resulting in enhanced EMT and promoted growth and metastasis of HCC. A HULC/USP22/Sirt1 protective autophagy pathway was further identified that attenuates HCC cell sensitivity to chemotherapeutic agents [52]. In the study, autophagy-related proteins were markedly increased upon HULC overexpression, but were significantly reduced upon Sirt-1 silencing. These results revealed a HULC-Sirt1-autophagy pathway, which was further discovered to be able to weaken the chemosensitivity of HCC cells toward oxaliplatin. As another example, the lncRNA X inactive-specific transcript (XIST) inhibits the proliferation of HCC cells through the miR-497-5p/PDCD4 or miR-92b/Smad7 pathway, but promotes HCC progression by silencing miR-194-5p and derepressing MAPK1 [92–94].

Together, these studies on the associations between lncRNAs and HCC show that lncRNAs function in a multidimensional network to influence HCC, not only by interacting with a large group of miRNAs and affecting their correlated pathways, but also by participating in a number of distinct mechanisms. Notably, some lncRNAs show both tumor suppressive and oncogenic functions, which calls for additional studies to determine how these seemingly contrary functions are balanced and regulated.

### CircRNAs as ceRNAs

CircRNAs are relatively more resistant to degradation compared with linear RNA because the 3′ and 5′ ends of circRNAs are covalently linked [101]. Apart from higher stability compared with linear RNAs, circRNAs are highly homologous to their linear counterparts and contain more MREs. Thus, circRNAs can act as effective miRNA sponges to disrupt miRNA-mediated target repression and regulate genes at both transcriptional and post-transcriptional levels as robust ceRNAs [102]. Emerging evidence has demonstrated that circRNAs may counteract miRNA-mediated repression of linear mRNAs by functioning as miRNA sponges (Table 3).

**Table 3:** Validated ceRNA networks shaped by the ceRNA function of circRNAs in HCC

ceRNA network type	ceRNA member	Shared miRNA(s)	Competing target (mRNA)	ceRNA role	Experiments related to mechanism	Related HCC function mechanisms	Ref
CircRNA–miRNA–mRNA	circASAP1	miR-326, miR-532-5p	MAPK1, CSF-1	Oncogenic	Overexpression, shRNA knockdown in mouse xenografts	Proliferation and invasion	[103]
	circCdr1as	miR-1270	AFP	Oncogenic	Cellular	Proliferation and migration	[104]
	circCdr1as	miR-7	CCNE1, PIK3CD	Oncogenic	Cellular	Proliferation and invasion	[105]
	circABCC2	miR-665	ABCC2	Oncogenic	siRNA knockdown in mouse xenografts	Proliferation and invasion	[106]
	circ_104718	miR-218-5p	TXNDC5	Oncogenic	overexpression, siRNA knockdown in mouse xenografts, miR mimics, miR inhibitors	Proliferation, migration and invasion	[107]
	circ_104075	miR-582-3p	YAP	Oncogenic	CRISPR/Cas9 knockout of upstream positive regulator in mouse models	Proliferation	[108]
	circFBLIM1	miR-346	FBLIM1	Oncogenic	siRNA knockdown in mouse xenografts	Proliferation and invasion	[109]
	circ_100338	miR-141-3p	RHEB	Oncogenic	Cellular	Migration and invasion	[110]
	circ_0067934	miR-1324	FZD5	Oncogenic	siRNA knockdown in mouse xenografts	Proliferation, migration and invasion	[111]
	circ_0078710	miR-31	HDAC, CDK2	Oncogenic	Overexpression in mouse xenografts	Proliferation, migration and invasion	[112]
	circ_ CDYL	miR-892a, miR-328-3p	HDGF, HIF1AN	Oncogenic	shRNA knockdown in mouse xenografts	Proliferation and chemoresistance	[113]
	circ_ DB	miR-34a	USP7	Oncogenic	Overexpression, shRNA knockdown in mouse xenografts	Proliferation	[114]
	circ_101368	miR-200a	HMGB1, RAGE	Oncogenic	Cellular	Migration and invasion	[115]
	circ_001569	miR-411-5p, miR-432-5p	–	Oncogenic	shRNA knockdown in mouse xenografts	Migration and invasion	[116]
	circ_0015756	miR-7	FAK	Oncogenic	shRNA knockdown in mouse xenografts	Proliferation, migration and invasion	[117]
	circRBM23	miR-138	CCND3	Oncogenic	Overexpression, siRNA knockdown in mouse xenografts	Proliferation and migration	[118]
	circ_0016788	miR-486	CDK4	Oncogenic	shRNA knockdown in mouse xenografts	Proliferation and invasion	[119]
	circ_0000673	miR-767-3p	SET	Oncogenic	siRNA knockdown in mouse xenografts	Proliferation and invasion	[120]
	circ_0005075	miR-431	-------	Oncogenic	Cellular	Proliferation, migration and invasion	[121]
		miR-335	MAPK1	Oncogenic	siRNA knockdown in mouse xenografts	Proliferation, migration and invasion	[122]
	circ_BIRC6	miR-3918	Bcl2	Oncogenic	Overexpression, shRNA knockdown in mouse xenografts	Proliferation, migration and invasion	[123]
	circ_ZNF652	miR-203, miR-502-5p	Snail	Oncogenic	Knockdown in mouse xenografts	EMT	[124]
	circ ZFR	miR-3619-5p	CTNNB1	Oncogenic	Cellular	Proliferation and EMT	[125]
	circ_0008450	miR-214-3p	EZH2	Oncogenic	siRNA knockdown in mouse xenografts	Proliferation, migration and invasion	[126]
	circ_0000517	miR‑326	SMAD6	Oncogenic	shRNA knockdown in mouse xenografts	Proliferation, colony formation, migration, and invasion	[127]
	circNFATC3	miR-548I	NFATC3	Tumor suppressive	shRNA knockdown in mouse xenografts	Proliferation, migration and invasion	[128]
	circ_103809	miR-620	-------	Tumor suppressive	Cellular	Proliferation, migration and invasion	[129]
	circC3P1	miR-4641	PCK1	Tumor suppressive	Overexpression in mouse xenografts, miR mimics	Proliferation, migration and invasion	[130]
	circ_0091570	miR-1307	ISM1	Tumor suppressive	Overexpression, siRNA knockdown in mouse xenografts	Proliferation and migration	[131]
	circ_0001649	miR-127-5p, miR-612, miR-4688	SHPRH	Tumor suppressive	shRNA knockdown in mouse xenografts	Proliferation and migration	[132]
	circMTO1	miR-9	p21	Tumor suppressive	siRNA knockdown in mouse xenografts	Proliferation and invasion	[133]
	cSMARCA5	miR-17-3p, miR-181b-5p	TIMP3	Tumor suppressive	Overexpression in mouse xenografts	Proliferation and migration	[134]
	circSETD3	miR-421	MAPK-14	Tumor suppressive	Overexpression in mouse xenografts	Proliferation	[135]
	circTRIM33-12	miR-191	TET1	Tumor suppressive	Overexpression, shRNA knockdown in mouse xenografts	Proliferation, migration, invasion and immune evasion	[136]
	circADAMTS13	miR-484	ADAMTS13	Tumor suppressive	Cellular	Proliferation	[137]
	circADAMTS14	miR-572	RCAN1	Tumor suppressive	Overexpression in mouse xenografts, miR mimics, miR inhibitors	Proliferation and invasion	[138]
	circSMAD2	miR-629	SMAD2	Tumor suppressive	Cellular	Migration, invasion and EMT	[139]
	circLARP4	miR-761	RUNX3	Tumor suppressive	shRNA knockdown in mouse xenografts	Proliferation	[140]
	circLARP4	miR-761	RUNX3	Tumor suppressive	shRNA knockdown in mouse xenografts	Proliferation	[140]
	circ_0005986	miR-129-5p	Notch1	Tumor suppressive	Cellular	Proliferation	[141]

ceRNA: competing endogenous RNA; circRNA: circularRNA; mRNAs: messenger RNAs; miRNA: microRNA; HCC: hepatocellular carcinoma; EMT: epithelial–mesenchymal transition; Ref: reference

Similar to other ceRNAs, circRNAs competitively inhibit miRNAs via their MREs. CircRNAs function in tumor signaling pathways through their regulation of cell proliferation, apoptosis, tumor invasion and migration. Qin et al. [142] demonstrated significantly decreased expression of hsa_circ_0001649 expression in HCC tissues (*P* = 0.0014), which was related to tumor embolus (*P* = 0.017) as well as tumor size (*P* = 0.045). Yao et al. [143] reported the significantly decreased expression of circZKSCAN1 (hsa_circ_0001727) in HCC (*P* < 0.05), which was associated with vascular invasion (*P* = 0.002) and cirrhosis (*P* = 0.031) as well as tumor number (*P* < 0.01). Hu et al. reported increased levels of circASAP1 in HCC patients with postoperative metastasis or recurrence [103]. Transplanted HCC mouse models verified that circASAP1 promoted proliferation as well as invasion of HCC through miR-326/miR-532-5p-MAPK signaling (Fig. 5b). Xenografts of tumors with shRNA-mediated knockdown of circASAP1 showed decreased numbers of metastatic pulmonary nodules, while the xenograft model derived from circASAP1-overexpressing cells had a higher rate of lung metastasis. In addition, circASAP1 enhanced the infiltration of tumor associated macrophages in xenografts, but no alterations of infiltrating neutrophils or fibroblasts were observed. Zhang et al. observed that overexpression of circSMAD2 attenuated migration, invasion and EMT of HCC by sponging miR-629 [139]. Taken together, these results provide evidence that circRNAs have a dual role in regulating tumor signaling pathways by competing with miRNAs with contrary functions.

CircRNAs are resistant to exonucleases, resulting in relatively stable expression and intracellular accumulation. Exosomal excretion is thus an important route of in vivo elimination of circRNAs. In a study by Su et al., SMMC-7721 and HepG2 cell-derived exosomal circRNA Cdr1 was transfected into 293T cells [104]. Consistent with its intracellular function, exosomal Cdr1as increased the proliferation and migration of 293T cells, indicating that circRNAs function in the cytoplasm and exosomes. Zhang et al. reported a similar finding in the circ-DB/miR-34a/USP7/Cyclin A2 pathway [114].

The activating receptor natural-killer group 2 member D (NKG2D) is a tumor-associated immune response marker, and its expression indicates immune response mediated by CD8 + T cells and γδ + T cells as well as NK cells in tumors. Zhang et al. proposed the circTRIM22-12/miR-191/TET1 pathway and validated it in circTRIM33-12-overexpressing nude mice xenograft models. circTRIM33-12 expression level was positively correlated with NKG2D-positive cell numbers in HCC, suggesting that circTRIM33-12 sponged miR-191/TET1 and exerted an anti-tumor function by not only participating in the tumor proliferation–related signaling pathway, but also contributing to immune surveillance and T cell activation [136].

Considering the high stability of circRNAs in body fluids, including blood and saliva, circRNAs are potential superior biomarkers for HCC diagnosis. The miR-328-3p/HIF1AN-NOTCH2 and miR-892a/HDGF-NCL-PI3K-AKT pathways regulated by circCDYL facilitate Survivin and Myc expression in HCC, suggesting that a combination of circCDYL, HIF1AN and HDGF can be used for the diagnosis of Barcelona Clinic Liver Cancer stage 0 and A of HCC [144]. CircRNA_104075, another potential diagnostic biomarker, was discovered to upregulate the expression of YAP by inhibiting miR-582-3p. Zhang et al. used the CRISPR/Cas9 strategy to establish a knockdown mouse model of HNF4a. HNF4a was formerly proved to be an HCC-promoting transcription promoter. HNF4a knockout mice were smaller in size, with a low liver weight, and had low expression of circ_104075, which indicates that circ_104075 transcription is stimulated by HNF4a. Intriguingly, an m6A modification in the 3′-untranslated region of YAP mRNA facilitates the interaction between YAP, miR-582-3p and circRNA_104075, demonstrating how circRNA function can be influenced by methylation or other modifications and the role of epigenetics in circRNA function [108].

Similar to lncRNAs, circRNAs exert their diverse functions in HCC through sponging miRNAs. CircRNAs exhibit multiple roles in different pathways and participate in HCC pathogenesis through various pathophysiologic processes. In addition, circRNAs show enhanced stability, making them promising candidates for novel cancer biomarkers.

### Pseudogenes as ceRNAs

Pseudogenes were previously described as non-functional, genomic junk DNAs sharing homology with the parental encoding sequence. Pseudogenes were characterized by a loss of protein-coding regions due to truncation or mutation and suggested to have no function. Transcriptomic and proteomic analyses have not only confirmed the ability of pseudogenes to produce transcripts and proteins, but also revealed novel miRNA/gene/pseudogene regulatory networks in cancer biology [145, 146]. Studies on the influence of pseudogenes in different types of tumors have revealed their roles in HCC (Table 4).

**Table 4:** Validated ceRNA networks shaped by the ceRNA function of pseudogene in HCC

ceRNA network type	ceRNA member	Shared miRNA(s)	Competing target (mRNA)	ceRNA role	Experiments related to mechanism	Related HCC function mechanisms	Ref
Pseudogene–miRNA–mRNA	OCT4-pg4	miR-145	OCT4	Oncogenic	siRNA knockdown in mouse xenografts	Proliferation and colony formation	[10]
	RACGAP1P	miR-15-5p	RACGAP1	Oncogenic	Overexpression in mouse xenografts	Proliferation and migration	[147]
	INTS6P1	miR-17-5p	INTS6	Tumor suppressive	Overexpression in mouse xenografts	Apoptosis and cell growth	[148]

ceRNA: competing endogenous RNA; mRNAs: messenger RNAs; miRNA: microRNA; HCC: hepatocellular carcinoma; Ref: reference

OCT4-pg4 is a pseudogene related to OCT4, a transcription factor that is involved in proliferation, pluripotency and self-renewal ability in embryonic stem cells and germ cells [149]. The expression of OCT4-pg4 is positively associated with OCT4, and both are increased in HCC. Wang et al. found that OCT4-pg4 serves as a molecular decoy towards miR-145 and prevents OCT4 inhibition, thus promoting HCC growth and tumorigenicity [10]. Survival analysis of 54 cases of HCC suggested a significant correlation between increased OCT4-pg4 expression levels and shortened overall survival as well as disease-free survival in HCC patients.

RACGAP, a member of the GTPase activation family, is an oncoprotein that enhances the proliferation and migration of HCC by activating the RhoA/ERK signaling pathway. The RACGAP gene is also the first gene reported as an independent biomarker for HCC recurrence. Wang et al. discovered the significant up-regulation of the RACGAP1P pseudogene in HCC, which was related to shortened survival, larger tumor size, elevated AFP level and advanced clinical stage [147]. Luciferase assays and in vivo assays demonstrated that miR-15-5p was sequestered from its endogenous target RACGAP by RACGAP1P, causing increased RACGAP expression and contributing to the RACGAP oncogenic network (Fig. 5c).

In addition to oncogenic pseudogenes, tumor suppressive pseudogenes have also been identified. The INTS6 pseudogene inhibits the tumor growth in several types of human cancers through G1 cell cycle arrest. Peng et al. showed that INTS6 inhibited HCC cell growth, migration and survival [148]. Furthermore, INTS6P1 facilitated tumor suppression by competing with oncogenic miR-17-5p. In vitro and in vivo assays also showed that both INTS6 and INTS6P inhibited HCC cell growth, migration and survival.

These studies indicate that pseudogenes serve as ceRNAs and affect tumorigenesis through ceRNETs. With respect to their role in HCC carcinogenesis, pseudogenes may be used as prognostic indicators, stratification factors or therapeutic targets, and these functions may lead to the development of precise and individualized therapeutic strategies for HCC. It is likely that HCC-associated pseudogenes acting as ceRNAs are rare. Nevertheless, with the widespread application of whole-genome high-throughput sequencing technology, we believe that more HCC-associated pseudogenes will be uncovered and applied in disease management.

## The clinical application of ncRNAs as ceRNAs in HCC

The current commonly used screening methods for HCC include the alpha-fetoprotein (AFP) diagnostic marker, ultrasonography, computed tomography, magnetic resonance imaging and liver biopsy [150]. Among these methods, imaging examination and serum AFP testing are the most common and basic screening approaches. However, even if a low-level cutoff is applied, the sensitivity value of AFP for diagnosing HCC is nearly 60% and the specificity is still inadequate [151]. Moreover, approximately 30% of patients with early-stage HCC cannot be detected using serum AFP as diagnostic biomarker [3]. According to the European Association for the Study of the Liver (EASL) and the American Association for the Study of Liver Diseases (AASLD) guidelines for HCC, serum AFP level can also be used to monitor HCC progression [152, 153]. Although the diagnostic rate of HCC has markedly increased from the adoption of serum AFP screening, the limited specificity of serum AFP has led to overdiagnosis and overtreatment [154]. In treating HCC patients with antineoplastic drugs, currently available regimens have shown disappointing results: both first-line therapy sorafenib and lenvatinib only prolong survival by nearly two months for patients with advanced-stage HCC [155]. Given the emerging evidence indicating that ncRNAs act as ceRNAs and suggesting their use as biomarkers and even therapeutic targets for HCC, further studies on ncRNAs may provide new insights and novel strategies for HCC diagnosis, surveillance and treatments.

### Potential diagnostic biomarkers in HCC

Several ncRNAs that act as ceRNAs have been identified in the circulation and other body fluids in HCC and other cancers [108, 156–158]. These results suggested the potential that ncRNAs that act as ceRNAs and their associated ceRNETs may be useful for clinical applications as diagnostic biomarkers of HCC (Table 5).

**Table 5:** Potential clinical application of ncRNAs function as ceRNAs in HCC

ncRNA as ceRNA	Location	Clinical application	Description	Ref
circRNA_104075^#^	Serum	Diagnosis	AUC: 0.973, Se: 96.0%, Sp: 98.3%	[108]
BCYRN 1*	Serum	Diagnosis, prognosis, potential therapeutic target	Diagnosis: AUC: 0.7834, Se: 62.10%, Sp: 90.54%; Prognosis: overexpression associates with worse prognosis	[159, 160]
lncRNA-RP11‐513I15.6, miR‐1262, RAB11A^†^	Serum	Diagnosis	AUC: NA, Se: 100%, Sp: 76.7%	[156]
OCT4-pg4^^^	Tissue	Prognosis, potential therapeutic target	Associates with prognosis	[10]
HOXD-AS1*	Tissue	Prognosis, potential therapeutic target	Associates with TNM stage and prognosis	[72]
SNHG6-003*	Tissue	Prognosis, potential therapeutic target	Associates with portal vein tumor thrombus, BCLC stage, distant metastasis and prognosis	[85]
AGAP2-AS1*	Tissue	Prognosis, potential therapeutic target	Associates with tumor size, histological grade, TNM stage, venous invasion and prognosis	[89]
CASC2*	Tissue	Prognosis, potential therapeutic target	Associates with venous infiltration, histological grade, TNM stage and prognosis	[98]
circRNA_104718^#^	Tissue	Prognosis, potential therapeutic target	Associates with vascular invasion and prognosis	[107]
circ_0000517^#^	Tissue	Prognosis, potential therapeutic target	Associated with tumor size, TNM stage, lymph node metastasis and prognosis	[127]
circNFATC3^#^	Tissue	Prognosis, potential therapeutic target	Associates with vascular invasion, histological grade and prognosis	[128]
MYCNOS, DLX6-AS1, LINC00221, CRNDE*	Tissue	Prognosis	Associates with prognosis	[161]
13 lncRNAs prognositc model^†^	Tissue	Prognosis	Associates with prognosis	[162]
3 lncRNAs and 6mRNAs prognostic model^†^	Tissue	Prognosis	Associates with prognosis	[163]

ncRNA: non-coding RNA; ceRNA: competing endogenous RNA; HCC: hepatocellular carcinoma; AUC: Area under the curve; Se: Sensitivity; Sp: Specificity; NA: Not available; BCLC: Barcelona Clinic Liver Cancer; Ref: reference

^*^ Long non-coding RNA; ^#^circular RNA; ^^^ pseudogene; ^†^ceRNA panel

Ming et al. demonstrated that the lncRNA BCYRN1 functions as a ceRNA to play a vital role in HCC [159]. The authors analyzed BCYRN1 expression in plasma from 124 HCC patients, 79 cirrhosis patients, 68 hepatitis B patients and 74 healthy controls. Plasma BCYRN1 expression of HCC patients was significantly higher than that of healthy controls (p < 0.001) or hepatitis B patients (p < 0.01) [159]. Furthermore, combined detection of BCYRN1 and AFP improved the diagnosis of HCC in their cohort. Their data also indicated that BCYRN1 in plasma performed well in HCC diagnosis.

Zhang et al. found that circ_104075 acts as ceRNA to sponge miR-582-3p to stimulate tumorigenesis via YAP [108]. Circ_104075 exhibited a sensitivity of 96.0% and a specificity of 98.3% in HCC diagnosis. Moreover, some recent studies revealed that panels consisting of ncRNAs and their related ceRNETs can also be used as diagnostic biomarkers for HCC. Asmaa et al. [156] constructed an lncRNA-associated ceRNET (lncRNA-RP11-513I15.6-miR1262-RAB11A mRNA) according to an in silico analysis, followed by validation in serum specimens and additional clinical and experimental assays. Receiver operating characteristic curve analysis revealed that RAB11A mRNA, lncRNA-RP11-513I15.6 and miR-1262 in the ceRNET were effective biomarkers to differentiate HCC subjects from healthy controls (area under curve: 0.963, 0.847 and 0.822, respectively). Moreover, the combination of serum exosomal miR-1262, lncRNARP11-513I15.6 and AFP measurements improved the diagnostic accuracy to detect HCC at the early stage to nearly 100% sensitivity and 76.7% accuracy [156]. With the development of specific algorithms for ncRNA detection and quantification, we believe that more and more ncRNAs that function as ceRNAs will be identified as diagnostic biomarkers for HCC.

### Potential prognostic biomarkers and therapeutic targets in HCC

Several studies have explored the clinical application of ncRNAs that function as ceRNA as prognostic biomarkers and potential therapeutic targets for HCC (Table 5). Cao et al. found that the lncRNA SNHG6-003 functions as a ceRNA to promote cell proliferation and induce drug resistance in HCC [85]. High expression of SNHG6-003 closely correlated with tumor progression and poor survival in HCC patients. The results suggested that targeting the ceRNA network involving SNHG6-003 may be a treatment strategy against HCC. Besides the tremendous diagnostic value in HCC, the prognostic value of BCYRN1 was also proven in a recent study. Ding et al. demonstrated that overexpression of BCYRN1 significantly expedited HCC cell growth, clone formation and movement abilities, while downregulation of BCYRN1 had the opposite effects [160]. The authors also found that BCYRN1 was overexpressed in HCC samples, which was associated with unfavorable prognosis in patients with HCC. The study findings indicated that BCYRN1, miR-490-3p, and POU3F2 formed a ceRNA mechanism to modulate the occurrence and progression of HCC, suggesting these can be served as potential target molecules for the management of HCC [160]. Recently, Lin et al. established a circRNA-miRNA-mRNA regulatory network by integrating the analysis of differentially expressed circRNAs, miRNAs and mRNAs in HCC [9]. The functional enrichment analysis of differentially expressed circRNA-related mRNAs, screened from the constructed HCC-associated ceRNETs, revealed that differentially expressed cirRNAs were significantly associated with cell proliferation, cell adhesion and cell migration. The differentially expressed circRNAs are involved in various processes including cell cycle, peroxisome proliferator-activated receptor signaling, chemical carcinogenesis and p53 signaling, playing vital roles in HCC progression. The refined circRNA-miRNA-mRNA regulatory modules associated with HCC carcinogenesis further uncovered three key circRNAs (hsa_circ_0004913, hsa_circ_0007456 and hsa_circ_0078279) that may play important roles in carcinogenesis and progression of HCC [9]. These circRNAs may competitively bind to miR-182 and miR-346 to affect their respective regulatory networks. Functionalization of previously uncharacterized transcripts could be achieved, partially by identifying the ceRNA interactors, to present a framework for predicting and validating ceRNA interactions; this strategy may be universally applied to any transcript. In a scale-free network, links between nodes follow a power-law distribution, which suggests that most nodes have only a few links, while a few nodes can possess a large number of links [164]. These post-transcriptional ceRNETs can be considered as scale-free networks, because their structure is constructed upon smaller interconnected subnetworks, in which the nodes (ceRNAs) are linked by a large number of connections (miRNAs) [11]. The few highly connected nodes, also known as target hubs, were found to be critically involved in biological processes [164]. Therefore, these critical nodes (three circRNAs and two miRNAs), identified by the constructed circRNA-associated ceRNETs, may represent ideal therapeutic targets for HCC.

As promising biomarkers in the screening, diagnosis and prognosis for HCC, ncRNAs as ceRNAs are also potential therapeutic targets. However, there are still significant gaps in our current understanding of ncRNA functions as ceRNAs and we are still far from being able to incorporate ncRNAs into clinical practice. Large-scale studies and clinical trials are required to validate the role of ncRNAs as ceRNAs in the clinical application for HCC.

## Conclusions

In this review, we briefly discussed ceRNAs as well as the principles and influencing factors of the interactions between ceRNAs, concentrating on the roles and molecular mechanisms of ncRNAs that function as ceRNAs in HCC. We also summarized the ideas and methods to study ceRNAs and ceRNETs in HCC and some commonly used databases.

Recently, significant progress has been made in the studies of ceRNAs in HCC. To date, studies on ncRNAs that act as ceRNAs in HCC have primarily involved overexpression and knockout assays in cells and animals. However, ceRNA activity is affected by other factors, including subcellular location and ceRNA component abundance, interactions with RNA binding proteins, RNA editing and ceRNA affinity in the endogenous cellular context. Whether the results shown in overexpression assays truly reflect the spontaneous ceRNA crosstalk during carcinogenesis in patients with HCC remains unknown. Therefore, more animal experiments and clinical trials should be performed to validate these results.

Additionally, the majority of identified ceRNA interactions reflect single binding partners, although emerging evidence indicates ceRNA crosstalk in large interconnected networks. Aside from direct interactions via shared miRNAs, secondary and indirect interactions might also exert significant effects on ceRNA modulation. Thus, further investigations of ceRNAs should not only concentrate on identifying binary ceRNA interactions but enroll network analyses of potential complex miRNA and ceRNA networks. Moreover, the scale-free network property of ceRNA regulation also poses a challenge in selecting HCC-related molecular therapeutic targets. Targeting nonessential nodes within regulatory networks could cause ineffective therapeutic responses, as cancer cells may overcome the resulting damage through alternative signaling pathways. Therefore, the selection of therapeutic targets situated in a hub position of a ceRNET should be considered in future screening studies for HCC therapeutic targets.

In summary, the recently developed research techniques and computational approaches as well as the continued uncovering of ceRNET components will facilitate more detailed studies of ncRNAs that act as ceRNAs in HCC. These findings will not only provide a more comprehensive understanding of the underlying mechanism of HCC pathogenesis and progression but also establish a novel direction for future strategies for the diagnosis, treatment and prevention of HCC.

## Data Availability

Not applicable.

## Abbreviations

HCC: Hepatocellular carcinoma
ceRNA: Competing endogenous RNA
ncRNA: Non-coding RNA
miRNA: MicroRNA
lncRNA: Long non-coding RNA
circRNA: Circular RNA
mRNA: Messenger RNA
MRE: MicroRNA response element
ceRNET: Competing endogenous RNA regulatory network
Ago: Argonaute
EMT: Epithelial-mesenchymal transition
AFP: Alpha-fetoprotein
AUC: Area under the curve
